# Cases of Cholera, with the Appearances on Dissection. Being an Extract from a Medical Report of the Diseases Which Occurred in the 16th Lancers, from June to September, 1823

**Published:** 1824-11

**Authors:** 


Art. VII.—*Cases of Cholera, with the Appearances on Dissection. Being an Extract from a Medical Report of the Diseases which occurred in the 16th Lancers, from June to September,* 1823.

Cholera.—Five were admitted under this head, and one taken ill whilst in hospital for a contusion: four died. The following are some of the cases, and appearances on dissection:

John Halford, aged twenty-eight; nine months in India; a musician; tall, and spare habit. Felt some sharp griping pain in his bowels at parade, on the morning of the 20th August; was obliged to dismount, but continued on parade until the troops were dismissed. On his return to his barrack, was smartly purged. Stomach sick, which was speedily followed with painful spasms of the abdominal muscles. The vomiting and purging continued incessant; he was quite overcome from the exertion of going to and from the privy, and fainted. In this state he was brought to hospital: thirty ounces of blood were taken away; and a bolus, consisting of one scruple of calomel and a grain of opium, given, and washed down with a draught of fifty drops of tinct. opii, in peppermint water. At half-past nine o'clock, both the vomiting and purging had abated; but the spasms continued, and extended to the gastrocnemii muscles. The pulse could not be felt at the wrist. The extremities and surface of the body were cold, and bedewed with a clammy sweat; the hands and fingers were shrivelled to an extraordinary degree, and of a pale purple colour; the face and surface of the whole body had a dirty, dark appearance; lips of a deep purple colour; the eyes were sunk into the sockets, and the cornea dull and heavy: in short, the countenance had undergone a most remarkable change, so that a person well acquainted with him could scarcely have recognized him. A full dose of calomel and opium was repeated, and friction with tinct. opii applied to the abdomen and extremities, whilst a warm bath was getting ready. At half-past ten he was put into the bath, and felt almost instantaneous relief from the spasms. There was a tendency to stupor; but he was quite clear and coherent in his replies, when his attention was roused. There was no return of vomiting or purging, from the time of admission. The spasms were not so severe, but returned frequently. Calomel was given freely, and at short intervals, in the state of powder, with or without tincture of opium and ether, according to the symptoms. A purgative enema, with turpentine, was given; but it produced no effect, and did not come away. Coma increased; he gradually became more and more exhausted, and died a few minutes after two o'clock P.M.

Absence of urgent thirst was the most remarkable circumstance in this case.

He took altogether seven scruple-doses of calomel, with three grains of opium and 160 drops of tinct. opii, and ether ʒjss.

*Appearances on Dissection.*—Great turgescence of the vessels of the head; slight effusion between the pia mater and tunica arachnoidea, on the right side of the middle lobe of the brain. In the ventricles, an unusual quantity of red serous fluid; and a much larger quantity at the base of the brain, and in the spinal canal.

The lower lobe of each lung in contact with the diaphragm was gorged with blood; the upper lobes were rather collapsed.

The pericardium had an unnatural red hue; the heart appeared small, and felt uncommonly firm, particularly the walls of the left ventricle: it offered great resistance to the scalpel; its texture appeared unnaturally dry and dense. The musculi pectonati were alike firm.

The coronary, and other superficial vessels of the stomach, were injected; but, externally, there was no appearance of inflammation of that organ. There were inflamed patches on different parts of the villous coat; and other parts of it presented an unnatural dark colour, which did not look like the effects of inflammation.

The convolutions of the small intestines showed an inflammatory blush; and the minute ramification of vessels on their surface, injected as they were, had a beautiful appearance.

The liver was uncommonly dark coloured, and its vessels gorged with blood. The coats of the gall-bladder appeared and felt thicker than natural.

There was nothing unusual in the quantity or appearance of the bile, and it flowed without interruption into the duodenum, on pressure being applied.

The porta was much distended.

Thomas Cornet, aged twenty-six years, had been six years in India; was awoke out of his sleep, about twelve o'clock at night, on the 26th of August, by a griping pain in his belly, which was succeeded by purging, vomiting, and spasms in the abdominal and gastrocnemii muscles. He patiently endured his sufferings until three o'clock, when he applied to his comrade, and a *dooley* was sent for, on which he reached the hospital at four A.M. He mentioned that he had been sleeping in the open air, and that he had been in the habit of doing so. The pulse could not be felt at the wrist; the extremities and surface of the body were cold; the purging and vomiting had moderated, but the spasms were very severe in the calves of the legs; thirst most urgent. A vein was opened, but no blood flowed. Opium and calomel were given in large doses. Frictions were used until a bath could be got ready. The first dose of medicine was immediately rejected; but afterwards, although there were frequent attempts to vomit, nothing was brought up, and all the succeeding doses were retained. The purging also ceased; and after six o'clock he suffered only from spasm and thirst. He was perfectly rational in his conversation. He staid only ten minutes in the bath.

At half-past seven A.M. he was drowsy, and appeared under the influence of the opium, but got no sleep. A new source of suffering now came on,—viz. a severe pain along the spine of the back. He continued tossing about, and calling for drink. Every movement he made brought on the spasms in the legs, and in the internal part of the right thigh. He was deaf to all entreaties, and, although perfectly sensible, could not keep quiet, or abstain from drink.

As the disease advanced, the head became more engaged, (perhaps the opium assisted;) he talked wildly, and about eleven A.M. laboured under a degree of coma. The calomel and frictions were persevered in, and a turpentine enema administered, and he had a watery dejection soon after. At three P.M. the stupor had increased considerably; the eyes and countenance had undergone a material change, and he had evidently lost ground. The enema was repeated, and calomel and frictions continued. He gradually became less sensible to the thirst and spasms. Coma increased, and he died at six P.M.

*Dissection.*—The scalp bled freely, on being detached from the cranium. All the blood-vessels on the surface and in the substance of the brain were gorged with blood, and there was a slight effusion of blood upon the inferior part of the right middle lobe. There was a great deal of water in the ventricles; it was limpid and colourless.

There was an unusual degree of vascularity both upon the internal and external sheath of the spinal marrow; and this was observable as far as the section was continued,—viz. to the fifth dorsal vertebra. The muscles of the back appeared more like a mass of coagulated blood than muscular fibres, their vessels were so gorged, and bled so freely.

The lungs were in the same state as Halford's; and the pericardium presented a like inflammatory blush, which was more remarkable on the inner surface.

The heart presented an appearance of disease almost indescribable. It seemed enlarged, was flabby, and flaccid in texture. Upon its surface there were many and remarkable morbid appearances, such as dark-coloured dots and blotches, on the right side, near the root of the pulmonary artery. The deviations from a healthy state were equally striking in the interior. Perhaps it would not be out of place to remark, that the man had been dead eighteen hours before dissection; whereas the former (Halford) had been dead less than four hours.

The convolutions of the small intestines presented exactly the same appearance as in Halford. The superficial vessels of the stomach were not unusually full; but, on raising it up, there was an appearance of increased vascularity internally, and the villous coat was found inflamed in several distinct patches; the intermediate spaces of an extraordinary dark colour. There was a quantity of calomel adhering to different parts of it. The stomach was not much distended, and principally contained the drink taken in; but the duodenum contained a thick fluid, of a dark and extraordinary colour, resembling hydrargyrum cum creta suspended in a thick fluid.

The liver appeared healthy; the gall-bladder moderately distended, and the duct pervious. The porta not much distended, as usual, and its blood appeared very dark and muddy. The spleen was very flaccid, and not of healthy structure.

It has been remarked above, that the dissection of this man was necessarily delayed till eighteen hours after death. The state of the body did not support the opinion that putrefaction sets in at a very early period in this disease: perhaps, the flaccidity of most of the viscera, which has been remarked, might in some degree be attributed to it; but the brain, which usually changes first, was in good preservation, and firm in texture.

